# A state-of-the-art technique to perform cloud-based semantic segmentation using deep learning 3D U-Net architecture

**DOI:** 10.1186/s12859-022-04794-9

**Published:** 2022-06-24

**Authors:** Zeeshan Shaukat, Qurat ul Ain Farooq, Shanshan Tu, Chuangbai Xiao, Saqib Ali

**Affiliations:** 1 Faculty of Information Technology, Beijing University of Technology, Beijing, People’s Republic of China; 2 Faculty of Environmental and Life Sciences, Beijing University of Technology, Beijing, People’s Republic of China; 3 Faculty of Computer Science, University of South Asia, Lahore, Pakistan

**Keywords:** Semantic segmentation, Deep learning, Cloud computing, Brain tumor, 3D U-Net

## Abstract

Glioma is the most aggressive and dangerous primary brain tumor with a survival time of less than 14 months. Segmentation of tumors is a necessary task in the image processing of the gliomas and is important for its timely diagnosis and starting a treatment. Using 3D U-net architecture to perform semantic segmentation on brain tumor dataset is at the core of deep learning. In this paper, we present a unique cloud-based 3D U-Net method to perform brain tumor segmentation using BRATS dataset. The system was effectively trained by using Adam optimization solver by utilizing multiple hyper parameters. We got an average dice score of 95% which makes our method the first cloud-based method to achieve maximum accuracy. The dice score is calculated by using Sørensen-Dice similarity coefficient. We also performed an extensive literature review of the brain tumor segmentation methods implemented in the last five years to get a state-of-the-art picture of well-known methodologies with a higher dice score. In comparison to the already implemented architectures, our method ranks on top in terms of accuracy in using a cloud-based 3D U-Net framework for glioma segmentation.

## Simple summary

Glioma is one of the most fatal type of cancer and it is necessary to find better detection and treatment options for it. Segmentation of a tumor is the most challenging task in identification various aspects and regions of the tumor. In current study, we developed a cloud-based 3D U-Net framework of brain tumor segmentation where the network can be trained for once and can be accessed from anywhere in the world using any terminal device. Our method performed brain tumor segmentation with a high accuracy score of 95% compared to the other state-of-the-art methodologies.

## Introduction

Brain tumors are the most dangerous type of tumors that causes life-threatening consequences. Glioma is the most common and aggressive primary brain tumor that comprises of 16% of neoplasms occurring in brain and central nervous system (CNS). Gliomas mostly occur in brain and 61% of all gliomas appear in the four lobes of the brain. However, they can also emerge in spinal cord, cerebellum, and brain stem [1, 2]. Glioma occurs usually at an age of 64 years on average but it can emerge at any time of life including childhood. It is a fatal type of cancer and the survival time of patients after diagnosis is less than 14 months on average [3]. According to World Health Organization, gliomas are classified into 4 grades depending on their malignancy i.e. grade I, grade II, grade III and grade IV. Grade I and grade II gliomas are considered as low-grade in which tumors grow slowly while grade III and grade IV are high-grade that grows quickly and can be fatal. Grade I gliomas occur rarely and are mostly limited to childhood. Grade II gliomas can appear at any age and mostly occur in young adults. Grade III and grade IV gliomas are the most malignant classes of brain tumor [4]. Among all the different types, glioblastoma is the most dangerous and malignant type with an incident rate of 3.2 in a population of 100,000 people. It spreads more quickly and it is difficult to remove it completely even after a surgery. An early and comprehensive diagnosis and treatment method is necessary for patient’s survival [5].

**Fig. 1:**
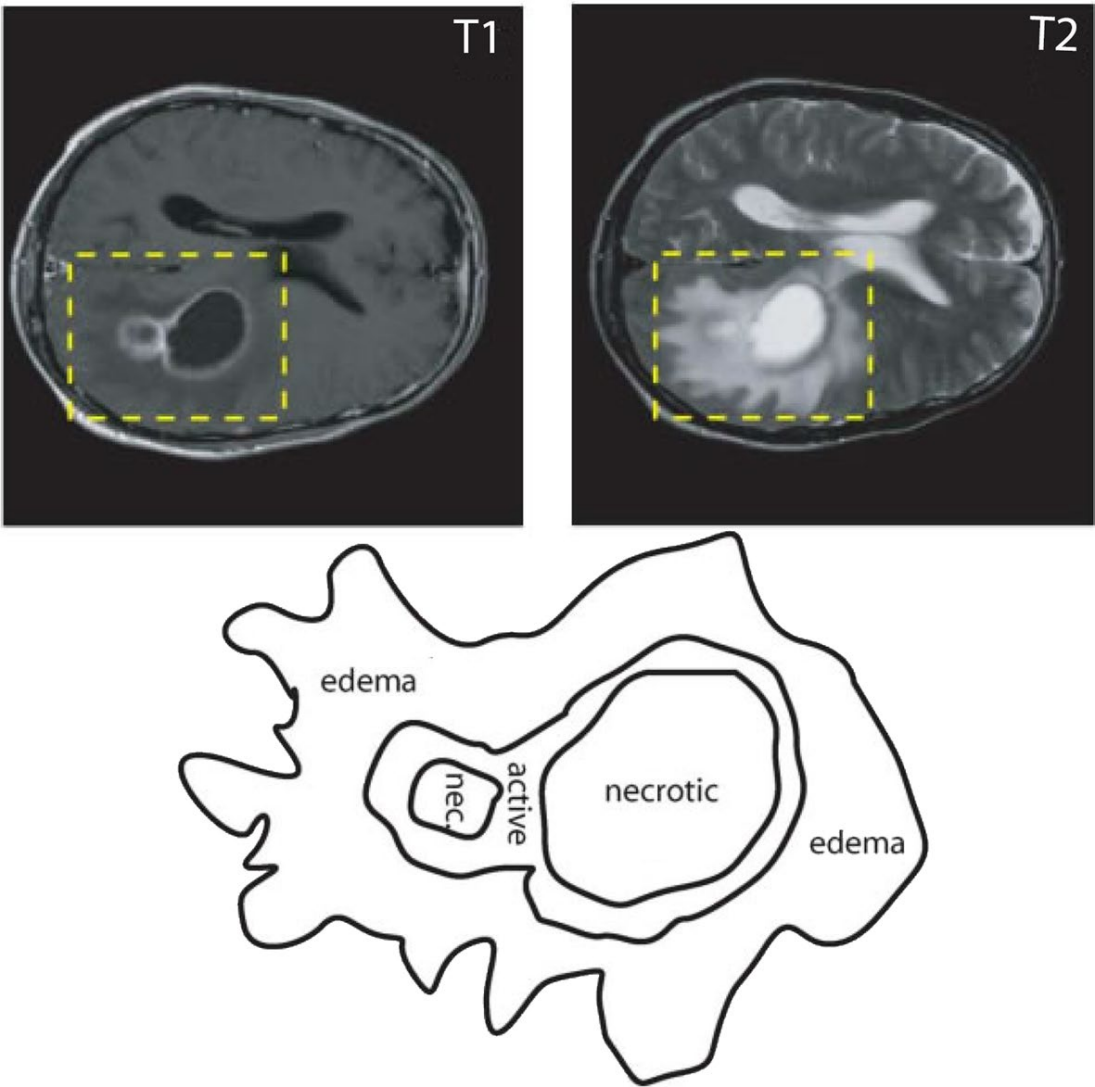
Segmentation showing different regions of tumor in a multimodality MRI image

Traditionally, medical imaging techniques such as Magnetic Resonance Imaging (MRI), Computer Tomography (CT), Positron Emission Tomography (PET), Single-Photon Emission Computed Tomography (SPECT), and Magnetic Resonance Spectroscopy (MRS) are used for diagnosis of brain tumors. Among these, MRI is considered as a standard technique for detection of tumors. MRI is a non-invasive procedure that excite the target cells and tissues by using radio frequency signals. It generates detailed internal images of the tissues and can also measure size, shape and localization of the tumor. There are four standard modalities of MRI used for the diagnosis of glioma that generates different type of images and yield important structural information of the tumors. The modalities are T1-weighted, T2-weighted, T1-weighted MRI with contrast enhancement (T1ce) and Fluid Attenuation Inversion Recovery (FLAIR). Each of these modalities represent various aspects of brain cells. T1 images focuses tumor contour and differentiates healthy tissues while T2 images show whole tumor region. Contrast enhanced T1-weighted MRI can easily distinguish tumor core regions due to the bright signals of the contrast agent. FLAIR images can differentiate between edema and Cerebrospinal Fluid (CSF). During an MRI acquisition, a 3D brain volume is represented by producing approximately 150 slices of 2D images [6]. Figure 1 showing Segmentation of different tumor regions in a multimodality MRI image.

Segmentation of tumors is a necessary task in the image processing of the gliomas and is important for starting a treatment. Manual segmentation of brain tumors is a time-consuming and laborious process and can only be performed by professional neuroradiologists. Compared to the manual segmentation, automatic techniques for brain tumor detection and segmentation are rapidly gaining interest and can assist in better diagnosis and treatment of brain tumor [6, 7].

During the past two decades, machine learning techniques have brought a rapid growth in classification and segmentation of brain tumors. The focus of most researches has been shifted on automatic segmentation of tumors using computer algorithms for quantitative assessment of brain tumors.

## Literature review

Zhao et al. [8] in 2017 proposed a novel brain tumor segmentation method by combining fully convolutional neural networks (FCNNs) and Conditional Random Fields (CRFs). They developed a unified framework and trained the deep learning model in 3 steps by using 2D image patches and image slices. BRATS 2013, BRATS 2015 and BRATS 2016 datasets were used. The experiment resulted in a better performance and it was concluded that unifying FCNNs and CRFs can improve the segmentation robustness. The dice score of the segmentation was compared with other competitive methods of brain tumor segmentation and it showed promising results in various ways. The method was ranked first among the 19 teams that participated in segmentation using BRATS 16 dataset for multi-temporal evaluation. Havaei et al. [9] presented a fully automatic brain tumor segmentation method using a novel architecture based on Convolutional Neural Networks (CNN). The dataset utilized in the study was BRATS 2013 and the results demonstrated that their proposed architecture gave improved results compared to the previously performed state-of-the-art methodologies. The method also proved to be 30 times faster than the other methods of brain tumor segmentation.

An efficient 11-layers deep, 3D Convolutional Neural Network (CNN) was proposed by Kamnitsas et al. [10], known as DeepMedic. The method analyzed the advantages of utilizing small convolutional kernels in 3D CNNs which resulted in a deeper and more discriminative network without any change in the computational cost. According to the author, their system achieved highest accuracy in brain lesion segmentation of patients with severe traumatic brain injury (TBI).

Zeineldin et al. [11] proposed DeepSeg, a generic deep learning architecture that utilized Fluid-attenuated inversion recovery (FLAIR) MRI data for automatic detection and segmentation of the brain lesion. The method applied different deep learning models and it accurately detected the tumor regions in the validated set with a mean dice score ranging from 0.81 to 0.84. The study utilized different CNN models including ResNet, DenseNet, and NASNet. Alkassar et al. [12] proposed a deep neural network (DNN) based architecture, SegNet for automatic segmentation of brain tumor using BRATS 2015 dataset. The framework consisted of three parts i.e. the encoder network, decoder network, and classification layer. Results of the experiment demonstrated that the proposed method achieved a global accuracy (0.9) and dice score (0.89) on BRATS 2015 dataset.

A novel approach for brain tumor segmentation was proposed by Chahal et al. [13] using Convolutional Neural Network (CNN) based models. The method utilized two types of architectures i.e. Input Cascade and Mean Failure Cost (MFC) Cascade. BRATS 2013 dataset was used containing MRI scans of both high grade (HG) and low grade (LG) gliomas. The resultant outcomes showed that the Input cascade gave better performance compared to the MFC cascade in both HG and LG glioma with a dice score of 0.943 and 0.950 respectively.

In another experiment, Ding et al. [14] proposed a novel architecture for multi-modal brain tumor segmentation which they named as “Deep Residual Dilate Network with Middle Supervision (RDM-Net)”. The framework integrates the residual network with dilated convolution and is designed to boost information propagation in representative pipelines. The method was evaluated on BRATS 215 benchmark dataset and in comparison, to other state-of-the-art methods, the framework showed better performance in terms of the dice score. Ramírez et al. [15] proposed a new Deep Variational Framework based on a U-Net CNN architecture for brain tumor segmentation. The framework allowed the CNN to optimize the parameters of the model using input data. BRATS 2015 Flair MRI dataset was used and the result showed promising outcomes of using the proposed framework with a dice score of 0.857.

A hybrid convolutional neural network architecture was proposed by Sajid et al.[16], which uses a patch-based approach for predicting output label by taking into account both local and contextual information. The method was tested on BRATS 2013 dataset containing different modalities of MRI. The dice score achieved by the method showed improved results when compared to other methodologies. Wang et al. [17] proposed a method called wide residual & pyramid pool network (WRN-PPNet) for automatic segmentation of glioma. In their method, 3D MRI images are used to get 2D slices which are then put into the WRN-PPNet model. The results of the experiment showed that the method is simple and can give a good dice score compared to the other state-of-the-art methods.

Jiang et al. [18] proposed a two-stage cascaded U-Net architecture for end-to-end multimodal brain tumor segmentation using BRATS 2019 challenge dataset. The resulting outcomes indicated that the proposed method achieved a good average dice score for the enhancing tumor, whole tumor and for the tumor core. Zhao et al. [19] performed useful tricks on Deep Convolution Neural Network (DCNN) to improve the accuracy of the method for brain tumor segmentation. They used BRATS 2019 dataset and performed different tricks on different levels including data processing, sampling, random patch-size training, semi-supervised learning and model devising. The method achieved a mean dice score of 0.81, 0.88 and 0.86 for enhancing tumor, whole tumor and core tumor respectively.

Yogananda et al. [20] developed a triple network architecture based on 3D-Dense-UNet for fully automatic brain tumor segmentation. The algorithm was tested on BRATS 2017, BRATS 2018 and an independent clinical data set from Oslo University Hospital. The results showed that the algorithm was one of the top 3 performers in segmenting tumor core on BRATS 2017 dataset and segmenting whole tumor and enhancing tumor on BRATS 2018 dataset.

Recently, Zhang et al. [21] performed automatic segmentation of glioma using an ensemble and post-processing. The aim of their study was to improve the segmentation performance. They reported that compared to the conventional CNN model, ensemble provides a more robust solution with less variance. The ensemble method takes advantages of three individual models which were 3D UNet, 3D MI-UNet, and joint 3D + 2D MI-UNet. Their implemented method scored an average dice score of 0.87.

In another research study, et al. [1] proposed a fully automatic brain tumor segmentation method that utilizes all the four MRI modalities. The techniques uses a Cascade Convolutional Neural Network (C-CNN) model that extracts both local and global features in two different routes. An average dice score of 0.90 was achieved using this approach.

## Deep Learning 3D U-Net architecture based on Cloud

**Fig. 2:**
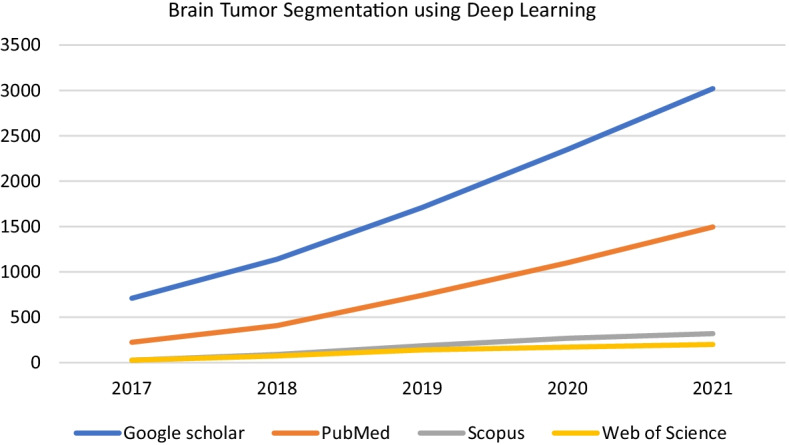
Published articles in Google Scholar, PubMed, Scopus and Web of Science in last 5 years

Deep learning methods have received huge attention of researchers for past few years and there has been a considerable increase in the amount of work done each year. We searched different databases to investigate the number of publications for the past 5 years in the area of brain tumor segmentation using deep learning and it showed a tremendous increase. Figure 2 shows a graph of number of publications in reputable web search engines/obliged databases including Google Scholar, PubMed, Scopus and Web of science with respect to their publication year.

In machine learning, appropriate representations are found out for the input data. Deep learning is a type of machine learning algorithm that learns a hierarchy of increasingly complex representations from the raw input [22]. For instance, it is used to detect sub tumoral region features across each candidate lesion using convolutional neural network (CNN) [23, 24]. CNNs are artificial neural networks that have multiple hidden convolutional layers and are capable of extracting higher level representative features [25]. Deep learning methods with CNN can perform various tasks related to medical imaging and have shown excellent results in skin cancer classification [26], diabetic retinopathy detection [27], and brain tumor segmentation [28–30]. Table 1 shows number of publications in reputable databases including Google Scholar, PubMed, Scopus and Web of science with respect to their publication year.

**Table 1:** Number of publications related to tumor Segmentation in last 5 years

Year	Google scholar	PubMed	Scopus	Web of science
2021	3020	1495	319	200
2020	2350	1100	268	170
2019	1710	742	185	140
2018	1140	408	89	74
2017	709	224	26	26

### Cloud based semantic segmentation using U-Net

Cloud computing came out as a powerhouse of enterprise technology [31]. The end users accelerate their application and begin working in a cloud computing network; they have no clue about the information of the servers, where they are located, or anything else [32]. Cloud computing is user-friendly and very advantageous; it provides services all over the world and permits anyone to utilize their services in a matter of minutes [33, 34].

U-Net is a simple network which is very efficient and fast, it has become popular in the semantic segmentation domain. In Semantic segmentation each pixel is labeled with a class in a voxel or image of a 3-D volume. This study illustrates the use of deep learning methods to perform binary semantic segmentation of brain tumors in magnetic resonance imaging (MRI) scans. In this binary segmentation, each pixel is labeled as tumor or background.

## State of art implementation

In image segmentation, a digital MRI image is partitioned into multiple divided segments with each segment having a distinct property. Traditionally, image segmentation helps in locating objects and boundaries in an image. In brain tumor segmentation, not only the location of the tumors is identified but extensions of the tumor regions including active tumorous tissue, necrotic or dead tissue, and edema (swelling near the tumor) are also detected [22]. Brain tumor segmentation identifies abnormal areas in the brain by comparing them with the normal tissues. Glioblastomas, the most malignant form of tumor infiltrate the neighboring tissues that causes unclear boundaries. Hence, they are hard to differentiate from normal tissues, as a result, multiple image modalities are used to identify glioblastomas. There are three sub categories of brain tumor segmentation based on the involvement of human which includes manual segmentation, semiautomatic segmentation, and fully automatic segmentation [35].

**Fig. 3:**
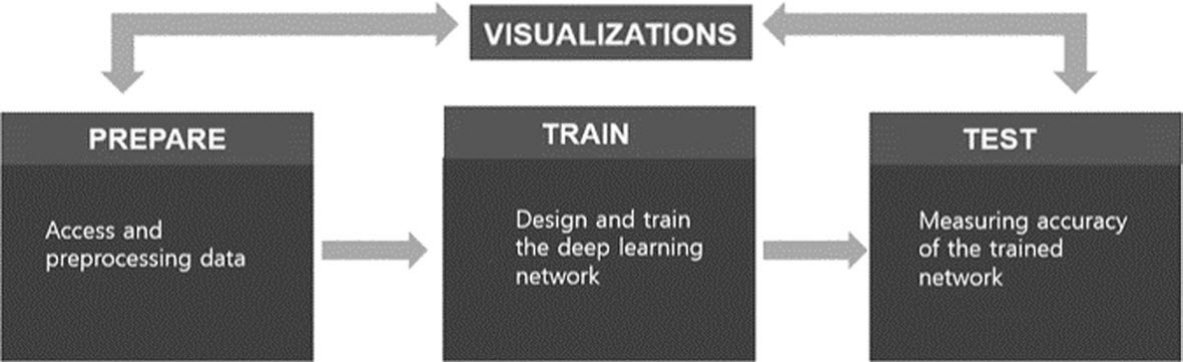
Semantic Segmentaion Workflow for Tumor MRIs

Manual segmentation involves the use of specialized tools by a human expert in drawing and painting the tumor regions and boundaries. Its accuracy depends upon the skills and knowledge of the operator performing it. Despite of the fact that manual segmentation is a laborious and time-consuming process, it is still considered as a gold standard for semi-automatic and fully automatic segmentation. Figure 3 shows workflow of brain tumor segmentation. In semi-automatic segmentation, human expertise and computer programs are combined and an operator is required to initialize the segmentation process and for evaluation of the results. Fully automatic segmentation does not require any human interaction. It involves the use of artificial intelligence in combination with prior knowledge and datasets to solve the segmentation problems [6].

Fully automatic brain tumor segmentation methods are classified into discriminative and generative methods. Discriminative methods usually depend upon supervised learning. Techniques that rely on supervised learning usually involve learning where the relationships between an image and a set of manually annotated data are learned from a huge dataset. In fully automatic image segmentation, machine learning algorithms has gained popularity due to their unmatched performance. Over the past few years, classical machine learning algorithms have been used extensively. However, due to the complexity of the data, classical machine learning techniques are not suitable for most applications [36]. Deep learning methods are becoming more popular due to their ability to learn and improve on complex computer vision tasks. Compared to discriminative methods, generative methods use prior knowledge such as location and size of the healthy tissues and generate probabilistic models [22].

### Expertimental setup

#### Available datasets

Automatic brain tumor segmentation has gained immense popularity in the past few years and there has been an increased interest in performing automatic brain tumor segmentation using publicly available datasets. The benchmark dataset Multi-modal Brain Tumor Image Segmentation (BRATS) [37], developed in 2012 is currently the most common publicly accessible dataset and has emerged to standardize performance evaluation in brain tumor segmentation process. Previously, The Internet Brain Segmentation Repository (IBSR) [38] and the BrainWeb datasets [39] have been used by several researchers in their image processing algorithms. The Reference Image Database to Evaluate Therapy Response (RIDER) [40] is another targeted data collection repository. RIDER neuro MRI contains imaging data of 19 patients with recurrent high-grade glioma and the dataset has been used by researchers in their automatic brain tumor segmentation experiments.

BRATS challenge contains datasets of four modalities T1, T1c, T2 and Flair belonging to both high-grade and low-grade gliomas. Initially, BRATS dataset contained only 30 MRI scans of glioma patients but the number grew substantially over the next few years. Medical Segmentation Decathlon (MSD) [41] is another challenge that provides a relatively larger dataset for brain tumor segmentation and can offer a wide range of modalities. It is actually a subset of the data of BRATS 2016 and 2017 challenge. It offers 750 multiparametric magnetic resonance images (mp-MRI) of both high and low-grade gliomas. The Decathlon challenge contains ten publicly available datasets that belongs to different regions of human body including brain, heart, hippocampus, liver, lung, pancreas, prostate, colon, hepatic vessel and spleen.

#### Dataset parameters for this study

**Fig. 4:**
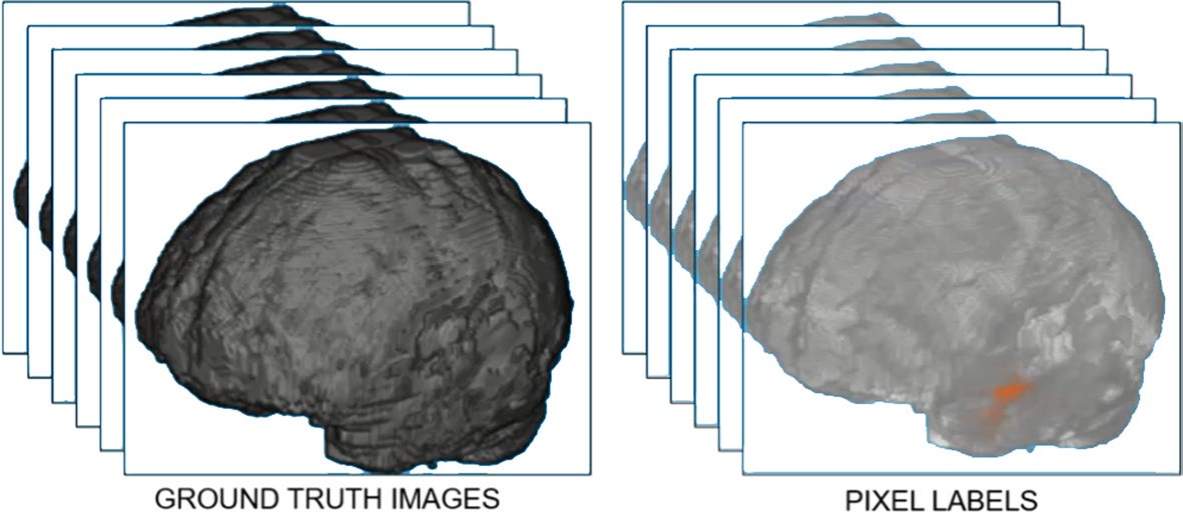
Dataset Ground Truth versus Pixel Labels

**Fig. 5:**
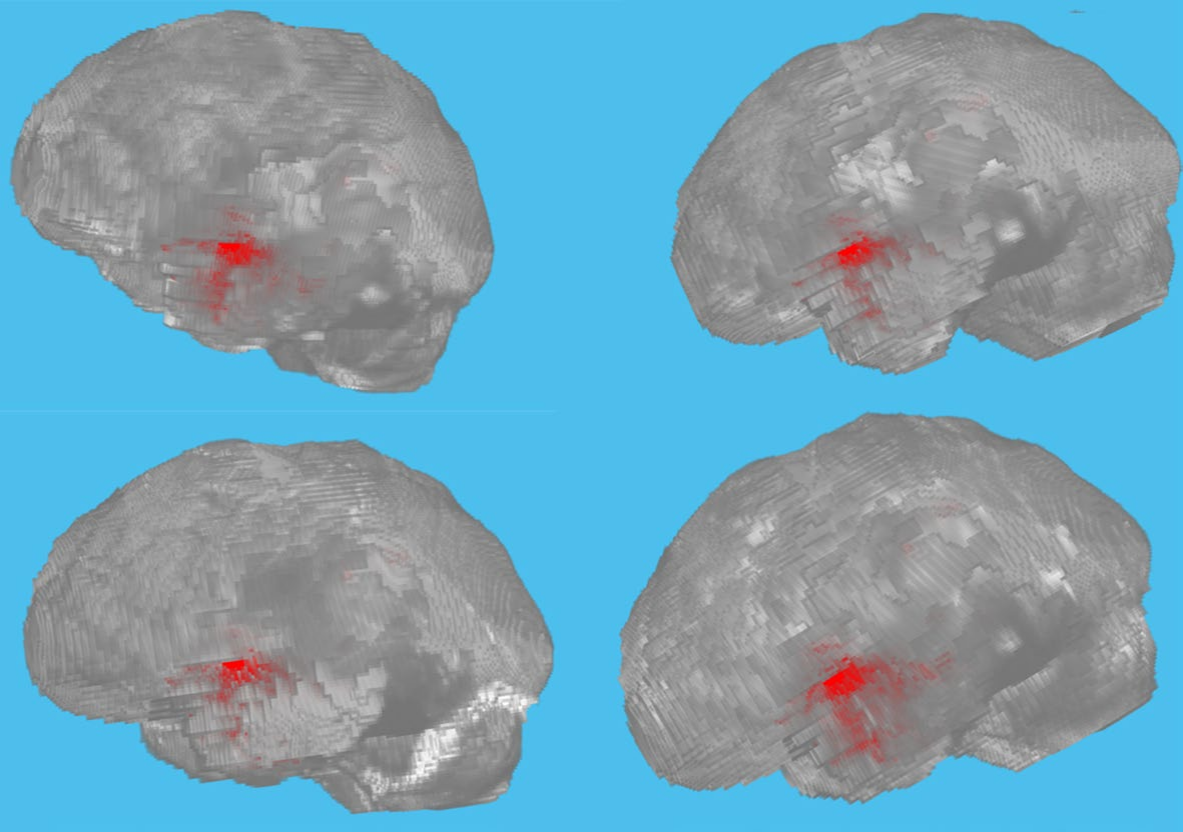
Preview of four different labeled training volumes from the dataset

We used the BraTS brain tumor dataset for training and validation. The size of the dataset was approximately 7 GB which contains 750(484 training volumes with voxel labels and 266 test volumes without labels) MRI scans of brain tumors as defined in Table 2, namely gliomas, having 4-D volumes, which represents 3-D images stack. Each 3-D volumetric image has dimensions 240(height) × 240(width) × 155(depth) × 4(different scan modalities). 484 training volumes were further divided into three independent sets that was used for training, testing and validation. Figure 4 showing volumetric image from dataset showing ground truth left as well labeled pixel on the right while Fig. 5 shows four different labeled training volumes.

**Table 2:** BraTS brain tumors dataset specifications

Target	Gliomas segmentation necrotic/active tumor and oedema	
Modality	Multimodal multisite MRI data	FLAIR
		T1w
		T1gd
		T2w
Size	750 4D volumes	484 training
		266 testing
Dimensions	Height	240
	Width	240
	Depth	155
	Dimen	Different scan modalities

### Experiment environment

We used Microsoft Azure Cloud Virtual Machine to run our experiment as it provides a low latency, high-throughput network interface optimized for tightly coupled parallel computing workloads. A CUDA Capable GPU is required for performing semantic segmentation of the image volumes. So, we choose N-series virtual machines as its ideal for compute and graphics-intensive workloads, like high-end remote visualization, deep learning, and predictive analytics, detailed experimental specifications are defined in Table 3. NC-series virtual machines feature the NVIDIA Tesla accelerated platform K80 GPU which dramatically lowers data center costs by delivering exceptional performance with fewer, more powerful cloud servers. It's engineered to boost throughput in real-world applications by 5-10x, while also saving customers up to 50% for an accelerated data center compared to a CPU-only system.

**Table 3:** Experimental specifications used to perform semantic segmentation on cloud

Cloud server	RAM	56 GiB
	Storage	340 GiB
	GPU	1 × K80
	Instance	Azure NC6
	vCPU(s)	6
	Cost	$1.321/hour
NVIDIA Tesla K80 accelerator	Memory	24 GB GDDR5
	Bandwidth	480 GB/s
	CUDA cores	4992
	Single-precision	8.73 teraflops
	Double-precision	2.91 teraflops

### Training and validation

#### Preprocessing

To efficiently train 3-D U-Net network, we need to preprocess the MRI dataset to crop it to a region primarily containing the brain and tumor. Cropping reduces the size of data as it only retains the critical part of each MRI volume and its corresponding labels. Each volume modality independently normalized by subtracting the mean and dividing by the standard deviation of the cropped region. Then the training volumes was further split into 55 test sets, 400 training sets, and 29 validation sets.

#### Random patch extraction

**Fig. 6:**
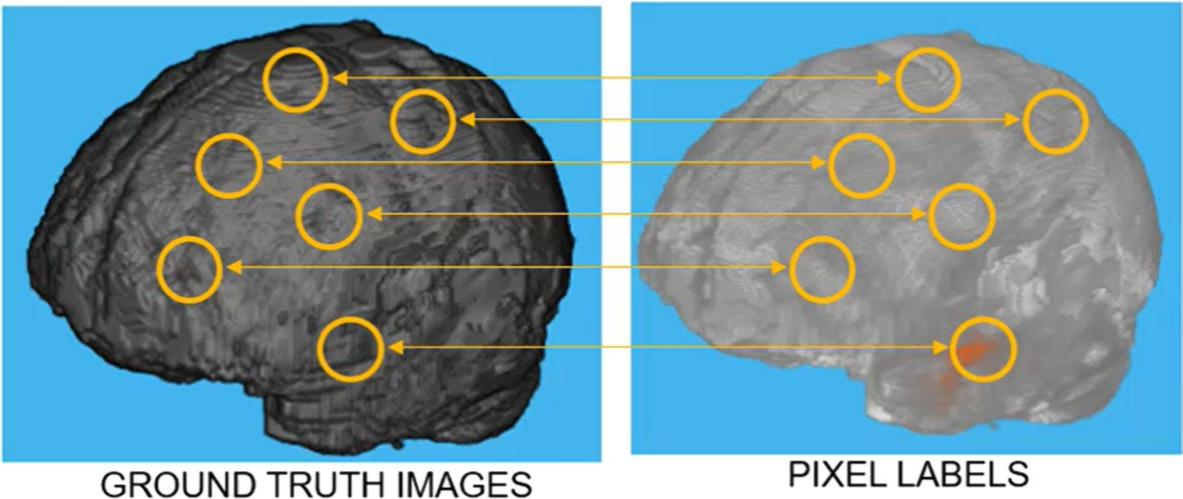
Random patch extraction datastore

Extracting Random Patches to prevent running out of memory is a common technique while training with large volumes as shown in Fig. 6. Use a random patch extraction datastore (specifications in Table 4) to feed the training data to the network and to validate the training progress. This datastore extracts random patches from ground truth images and corresponding pixel label data.

**Table 4:** Random patch extraction datastore specifications

Patch size	64 × 64 × 64 voxels
Patch per image	16
Mini-batch size	8

To make the training more robust, we used a function to augment 3D Patch which randomly reflects and rotates the training data. As time progresses, to evaluate whether the network is continuously learning, underfitting, or overfitting we used validation data.

### 3-D U-Net layers set up

This study practices discrepancy of the 3-D U-Net network as in U-Net, the preliminary sequences of convolutional layers (CL) are intermingled with max pooling layers, consecutively reducing the resolution of the input image. These layers are trailed by a sequence of convolutional layers intermingled with upsampling operators, consecutively increasing the resolution of the input image. The zero padding convolutions returns the same output size as of input.

Deep Learning 3-D U-Net using following layers:

- 3-D image input layer
- 3-D convolution layer for convolutional neural networks
- Batch normalization layer
- Leaky rectified linear unit layer
- 3-D max pooling layer
- Transposed 3-D convolution layer
- Softmax output layer
- Concatenation layer

The first, image Input 3d Layer, operates on image patches of size 64 × 64 × 64 voxels. The image input layer in 3-D U-Net is trailed by the contracting path, which consists of three encoder modules. Each encoder contains two convolution layers with 3 × 3 × 3 filters that double the number of feature maps, followed by a nonlinear activation using reLu layer. The first convolution is also followed by a batch normalization layer. Each encoder ends with a max pooling layer that halves the image resolution in each dimension.Unique names assigned to all the layers in the network.

For example, "en1" denotes the first encoder module and "de4" denotes the fourth decoder module. Where “en” denotes to encoder and “de” denotes to decoder while 1 and 4 are corresponding index to that module.

**Fig. 7:**
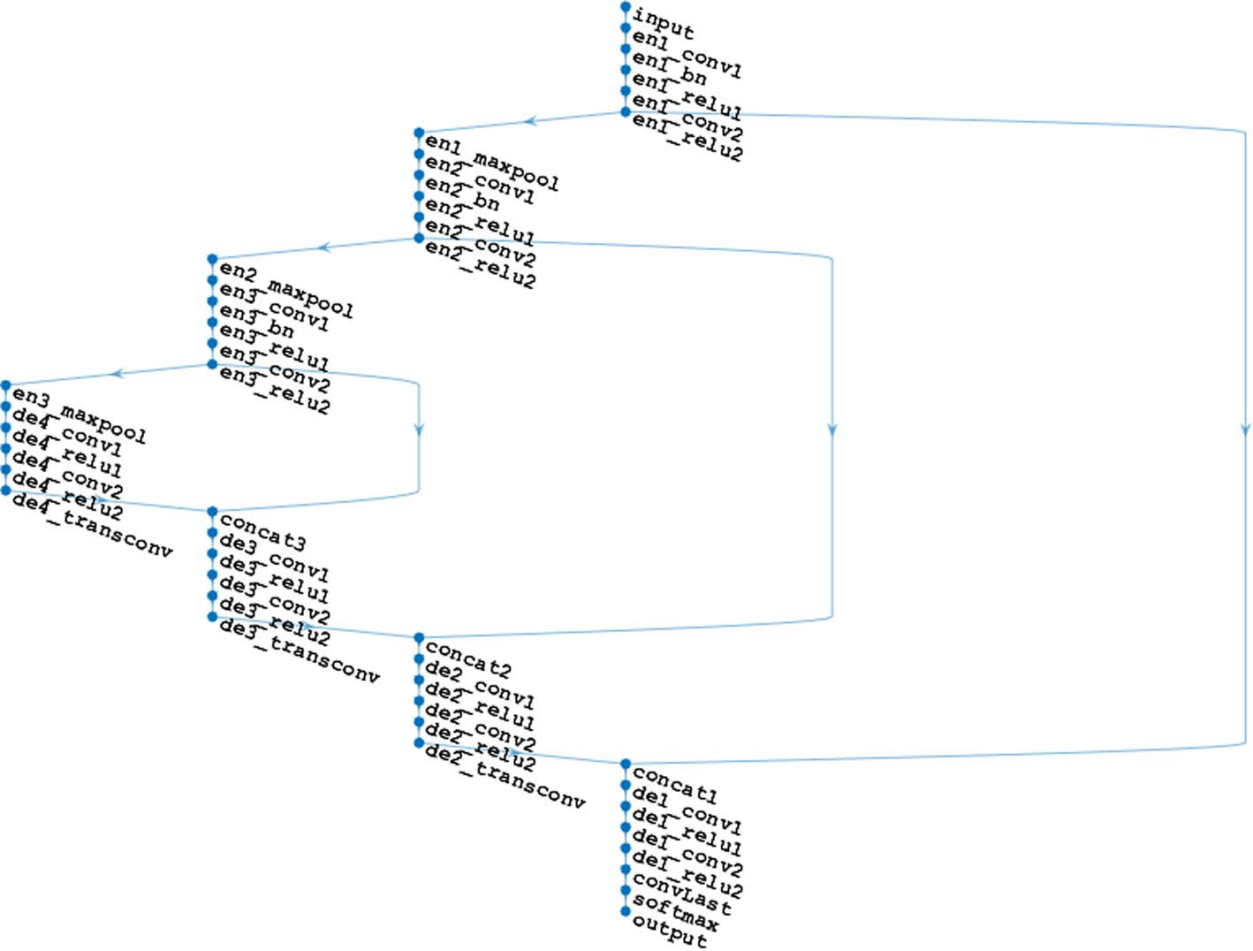
3-D U-Net Layers Diagram

**Fig. 8:**
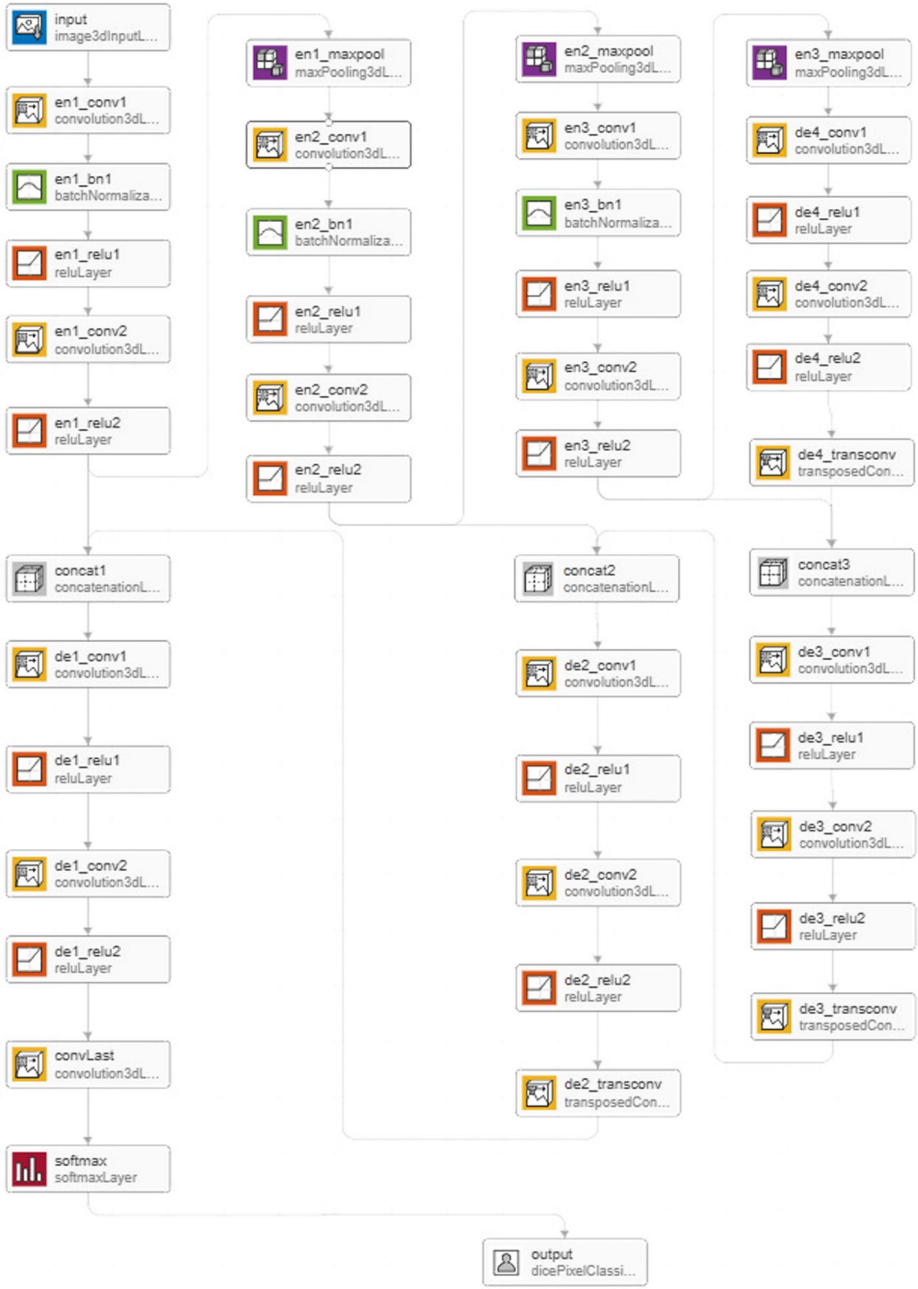
3D U-Net Deep Network Diagram used to train the system

The expanding path were created of the 3-D U-Net which consists of four decoder modules as shown in Fig. 7, while Fig. 8 shows 3D U-Net Deep Network diagram we used to train the system. The result analysis is given in Table 5 below. All decoders comprise of two convolution layers with same filters as of encoder that halve the number of feature maps, trailed by a nonlinear activation using a reLu layer. The first three decoders conclude with a transposed convolution layer that upsamples the image by a factor of 2. The final decoder includes a convolution layer that maps the feature vector of each voxel to the classes.

**Table 5:** Network analysis result of 3D U-net layers with reference to Figs. 7 and 8

Sr	Name	Type	Activations	Learnable	Total learnable
1	Input 64 × 64 × 64 × 4 images	3-D Image Input	64 × 64 × 64 × 4	–	0
2	en1_conv1 32 3 × 3 × 3 × 4 convolution with stride [1 1 1] and padding ‘same’	Convolution	64 × 64 × 64 × 32	Weights 3 × 3 × 3 × 4 × 32 Bias 1 × 1 × 1 × 32	3488
3	en1_bn1 Batch normalization with 32 channels	Batch Normalization	64 × 64 × 64 × 32	Offset 1 × 1 × 1 × 32 Scale 1 × 1 × 1 × 32	64
4	en1_relu1 ReLU	ReLU	64 × 64 × 64 × 32	–	0
5	en1_conv2 64 3 × 3 × 3 × 32 convolution with stride [1 1 1] and padding ‘same’	Convolution	64 × 64 × 64 × 64	Weights 3 × 3 × 3 × 32 × 64 Bias 1 × 1 × 1 × 64	55,360
6	en1_relu2 ReLU	ReLU	64 × 64 × 64 × 64	–	0
7	en1_maxpool 2 × 2 × 2 max pooling with stride [2 2 2] and padding ‘same’	3-D Max Pooling	32 × 32 × 32 × 64	–	0
8	en2_conv1 64 3 × 3 × 3 × 64 convolution with stride [1 1 1] and padding ‘same’	Convolution	32 × 32 × 32 × 64	Weights 3 × 3 × 3 × 64 × 64 Bias 1 × 1 × 1 × 64	110,656
9	en2_bn1 Batch normalization with 64 channels	Batch Normalization	32 × 32 × 32 × 64	Offset 1 × 1 × 1 × 64 Scale 1 × 1 × 1 × 64	128
10	en2_relu1 ReLU	ReLU	32 × 32 × 32 × 64	–	0
11	en2_conv2 128 3 × 3 × 3 × 64 convolution with stride [1 1 1] and padding ‘same’	Convolution	32 × 32 × 32 × 128	Weights 3 × 3 × 3 × 64 × 128 Bias 1 × 1 × 1 × 128	221,312
12	en2_relu2 ReLU	ReLU	32 × 32 × 32 × 128	–	0
13	en2_maxpool 2 × 2 × 2 max pooling with stride [2 2 2] and padding ‘same’	3-D Max Pooling	16 × 16 × 16 × 128	–	0
14	en3_conv1 128 3 × 3 × 3 × 128 convolution with stride [1 1 1] and padding ‘same’	Convolution	16 × 16 × 16 × 128	Weights 3 × 3 × 3 × 128 × 128 Bias 1 × 1 × 1 × 128	442,496
15	en3_bn1 Batch normalization with 128 channels	Batch Normalization	16 × 16 × 16 × 128	Offset 1 × 1 × 1 × 128 Scale 1 × 1 × 1 × 128	256
16	en3_relu1 ReLU	ReLU	16 × 16 × 16 × 128	–	0
17	en3_conv2 256 3 × 3 × 3 × 128 convolution with stride [1 1 1] and padding ‘same’	Convolution	16 × 16 × 16 × 256	Weights 3 × 3 × 3 × 128 × 256 Bias 1 × 1 × 1 × 256	884,992
18	en3_relu2 ReLU	ReLU	16 × 16 × 16 × 256	–	0
19	en3_maxpool 2 × 2 × 2 max pooling with stride [2 2 2] and padding ‘same’	3-D Max Pooling	8 × 8 × 8 × 256	–	0
20	de4_conv1 256 3 × 3 × 3 × 256 convolution with stride [1 1 1] and padding ‘same’	Convolution	8 × 8 × 8 × 256	Weights 3 × 3 × 3 × 256 × 256 Bias 1 × 1 × 1 × 256	1,769,728
21	de4_relu1 ReLU	ReLU	8 × 8 × 8 × 256	–	0
22	de4_conv2 512 3 × 3 × 3 × 256 convolution with stride [1 1 1] and padding ‘same’	Convolution	8 × 8 × 8 × 512	Weights 3 × 3 × 3 × 256 × 512 Bias 1 × 1 × 1 × 512	3,539,456
23	de4_relu2 ReLU	ReLU	8 × 8 × 8 × 512	–	0
24	de4_transconv 512 2 × 2 × 2 × 512 transposed 3D convolutions with stride [2 2 2] and cropping [0 0 0; 0 0 0]	Transposed Convolution 3D	16 × 16 × 16 × 512	Weights 2 × 2 × 2 × 512 × 512 Bias 1 × 1 × 1 × 512	2,097,664
25	concat3 Concatenation of 2 inputs along dimension 4	Concatenation	16 × 16 × 16 × 768	–	0
26	de3_conv1 256 3 × 3 × 3 × 768 convolution with stride [1 1 1] and padding ‘same’	Convolution	16 × 16 × 16 × 256	Weights 3 × 3 × 3 × 758 × 256 Bias 1 × 1 × 1 × 256	5,308,672
27	de3_relu1 ReLU	ReLU	16 × 16 × 16 × 256	–	0
28	de3_conv2 256 3 × 3 × 3 × 256 convolution with stride [1 1 1] and padding ‘same’	Convolution	16 × 16 × 16 × 256	Weights 3 × 3 × 3 × 256 × 256 Bias 1 × 1 × 1 × 256	1,769,728
29	de3_relu2 ReLU	ReLU	16 × 16 × 16 × 256	–	0
30	de3_transconv 256 2 × 2 × 2 × 256 transposed 3D convolutions with stride [2 2 2] and cropping [0 0 0; 0 0 0]	Transposed Convolution 3D	32 × 32 × 32 × 256	Weights 2 × 2 × 2 × 256 × 256 Bias 1 × 1 × 1 × 256	524,544
31	concat2 Concatenation of 2 inputs along dimension 4	Concatenation	32 × 32 × 32 × 384	–	0
32	de2_conv1 128 3 × 3 × 3 × 384 convolution with stride [1 1 1] and padding ‘same’	Convolution	32 × 32 × 32 × 128	Weights 3 × 3 × 3 × 384 × 128 Bias 1 × 1 × 1 × 128	1,327,232
33	de2_relu1 ReLU	ReLU	32 × 32 × 32 × 128	–	0
34	de2_conv2 128 3 × 3 × 3 × 128 convolution with stride [1 1 1] and padding ‘same’	Convolution	32 × 32 × 32 × 128	Weights 3 × 3 × 3 × 128 × 128 Bias 1 × 1 × 1 × 128	442,496
35	de2_relu2 ReLU	ReLU	32 × 32 × 32 × 128	–	0
36	de2_transconv 128 2 × 2 × 2 × 128 transposed 3D convolutions with stride [2 2 2] and cropping [0 0 0; 0 0 0]	Transposed Convolution 3D	64 × 64 × 64 × 128	Weights 2 × 2 × 2 × 128 × 128 Bias 1 × 1 × 1 × 128	131,200
37	concat1 Concatenation of 2 inputs along dimension 4	Concatenation	64 × 64 × 64 × 192	–	0
38	de1_conv1 64 3 × 3 × 3 × 192 convolution with stride [1 1 1] and padding ‘same’	Convolution	64 × 64 × 64 × 64	Weights 3 × 3 × 3 × 192 × 64 Bias 1 × 1 × 1 × 64	331,840
39	de1_relu1 ReLU	ReLU	64 × 64 × 64 × 64	–	0
40	de1_conv2 64 3 × 3 × 3 × 64 convolution with stride [1 1 1] and padding ‘same’	Convolution	64 × 64 × 64 × 64	Weights 3 × 3 × 3 × 64 × 64 Bias 1 × 1 × 1 × 64	110,656
41	de1_relu2 ReLU	ReLU	64 × 64 × 64 × 64	–	0
42	convlast 2 1 × 1 × 1 × 64 convolution with stride [1 1 1] and padding ‘same’	Convolution	64 × 64 × 64 × 2	Weights 1 × 1 × 1 × 64 × 2 Bias 1 × 1 × 1 × 2	130
43	softmax softmax	Softmax	64 × 64 × 64 × 2	–	0
44	Output Dice loss	Classification Output	–	–	0

The concluding decoder consist of a convolution layer that maps the feature vector of each voxel to each of the two classes (background and tumor region). The custom Dice pixel classification layer weights the loss function to increase the effect of the small tumor regions on the Dice score.

Dice loss is calculated by using Sørensen-Dice similarity coefficient which measures the overlap between two segmented volumes. The general Dice loss L between one image Y and the corresponding T (ground truth) is given by

1 $$L = 1 - \frac{{2\sum\nolimits_{k = 1}^{K} {w_{k} \sum\nolimits_{m = 1}^{M} {Y_{km} T_{km} } } }}{{\sum\nolimits_{k = 1}^{K} {w_{k} \sum\nolimits_{m = 1}^{M} {Y_{km}^{2} + T_{km}^{2} } } }}$$

where M is the number of elements along the first two dimensions of Y(image), K is the number of classes and W_k_ is a class specific weighting factor that controls the influence each class makes to the loss. W_k_ is characteristically the opposite area of the expected region:

2 $$W_{k} = \frac{1}{{\left( {\sum\nolimits_{{m = 1}}^{M} {T_{{km}} } } \right)^{2} }}$$

This weighting used to reduce the influence of larger regions on the Dice score making it easier for the network to learn how to segment smaller regions. Concatenatin were done using input layer and encoder modules with the fourth decoder module. While other decoder modules were added as separate branches to layer graph. Concatenation Layers were used to connect the second reLu layer of each encoder module with a transposed convolution layer of equal size from a decoder module. The output of each concatenation layer were connected to the first convolution layer of the decoder module.

To effectively train the system "Adam" optimization solver was used with following hyperparameters shown in Table 6.

**Table 6:** System parameters to train 3D U-Net network

Initial learning rate	5e-4
Maxepochs	100
Learning rate schedule	Piecewise
Learning rate drop period	5
Learning rate drop factor	0.95
Validation frequency	400
Verbose	False
Mini-batch size	8

Methemtical experenseion of algorithm to effectively train the system can be defined as.

3 $$m_{t} = \beta_{1} m_{t - 1} + (1 - \beta_{1} )\left[ {\frac{\delta L}{{\delta w_{t} }}} \right]v_{t} = \beta_{2} v_{t - 1} + (1 - \beta_{2} )\left[ {\frac{\delta L}{{\delta w_{t} }}} \right]^{2}$$

In Eq. 3 m_t_ describes aggregate of gradients at time t, V_t_ denotes sum of square of past gradients. While W_t_ is weights at time t, ∂L is derivative of Loss Function and ∂W_t_ is derivative of weights at time t, β denotes Moving average parameter, ϵ is equal to a small positive constant.”

## Results

**Fig. 9:**
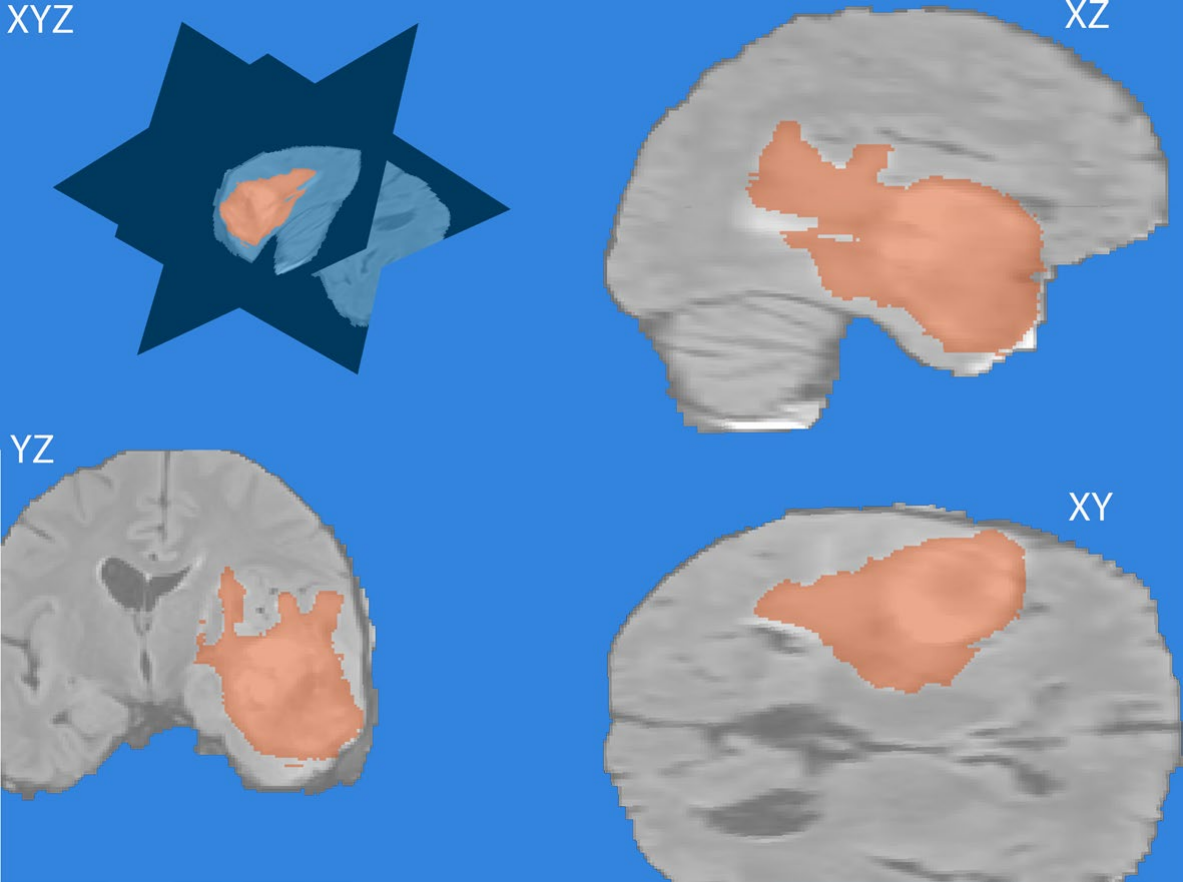
3D Orientation at Corresponding angles of a Labeled Volume

Gliomas are the most common primary brain malignancies. Our method identified tumor with a high accuracy. We obtained an average DSC of 0.95583/95% across the test set which is higher than the other cloud-based brain tumor segmentation methods using deep learning. Figure 9 below shows 3D orientation at corresponding angles of a Labeled Volume.

An extensive review was performed to compare our method with other state-of-the-art methods for semantic segmentation of brain tumor. Table 7 shows the list of studies performed in the last five years with a high accuracy and dice score. All of the studies used different architectures based on deep learning. A lot of work has been performed on brain tumor segmentation in the last few years and various researchers have proposed different architectures to obtain the maximum accuracy and dice score. It was not possible to include the details of all of the work done on brain tumor segmentation in this paper but we chose some of them with well-represented architectures and better results.

**Table 7:** List of methods with a high accuracy and dice score

Dataset	Segmentation method	Dice score	Publication year	References
BRATS 2013 BRATS 2015	FCNNs and CRFs	0.83 0.82	2017	[8]
BRATS 2013	CNN	0.80	2017	[9]
BRATS 2015	DeepMedic + CRF	0.71	2017	[10]
BRATS 2019	CNN	0.84	2020	[11]
BRATS 2015	FCN	0.89	2019	[12]
BRATS 2013	CNN	HG 0.943 LG 0.950	2019	[13]
BRATS 2015	RDM-Net	0.73	2019	[14]
BRATS 2015	CNN + TVS	0.857	2018	[15]
BRATS 2013	Hybrid CNN	0.86	2019	[16]
BRATS 2015	WRN-PPNet	0.94	2019	[17]
BRATS 2019	Two-stage Unet	0.84	2020	[18]
BRATS 2019	DNN	0.85	2020	[19]
BRATS 2017 BRATS 2018 Oslo Dataset	3D U-Net	0.82 0.84 0.80	2020	[20]
BRATS 2020	Ensemble + post-processing	0.87	2021	[21]
BRATS 2018	C-ConvNet	0.90	2021	[1]
BRATS 2020	3D U-NET	0.95	2022	This Study

**Fig. 10:**
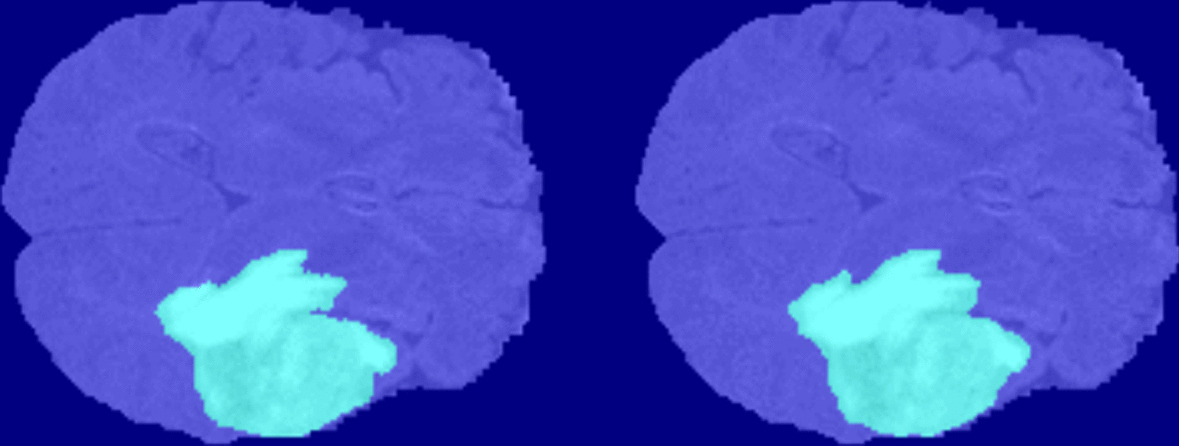
Labled Ground Truth Left versus Network Predicted Right

Table 8 shows the Summary of the evaluation metrics commonly used for Brats datasets. With respect to the number of false positive (FP), true positive (TP), false negative (FN) and true negative (TN) (Fig. 10).

**Table 8:** Summary of evaluation matrics

Metrics	Expression
Dice score (DSC)	$DSC = \frac{2TP}{{2TP + FP + FN}}$
Sensitivity (SEN)	$SEN = \frac{TP}{{TP + FN}}$
Specificity (SPEC)	$SPEC = \frac{TN}{{TN + FP}}$

**Fig. 11:**
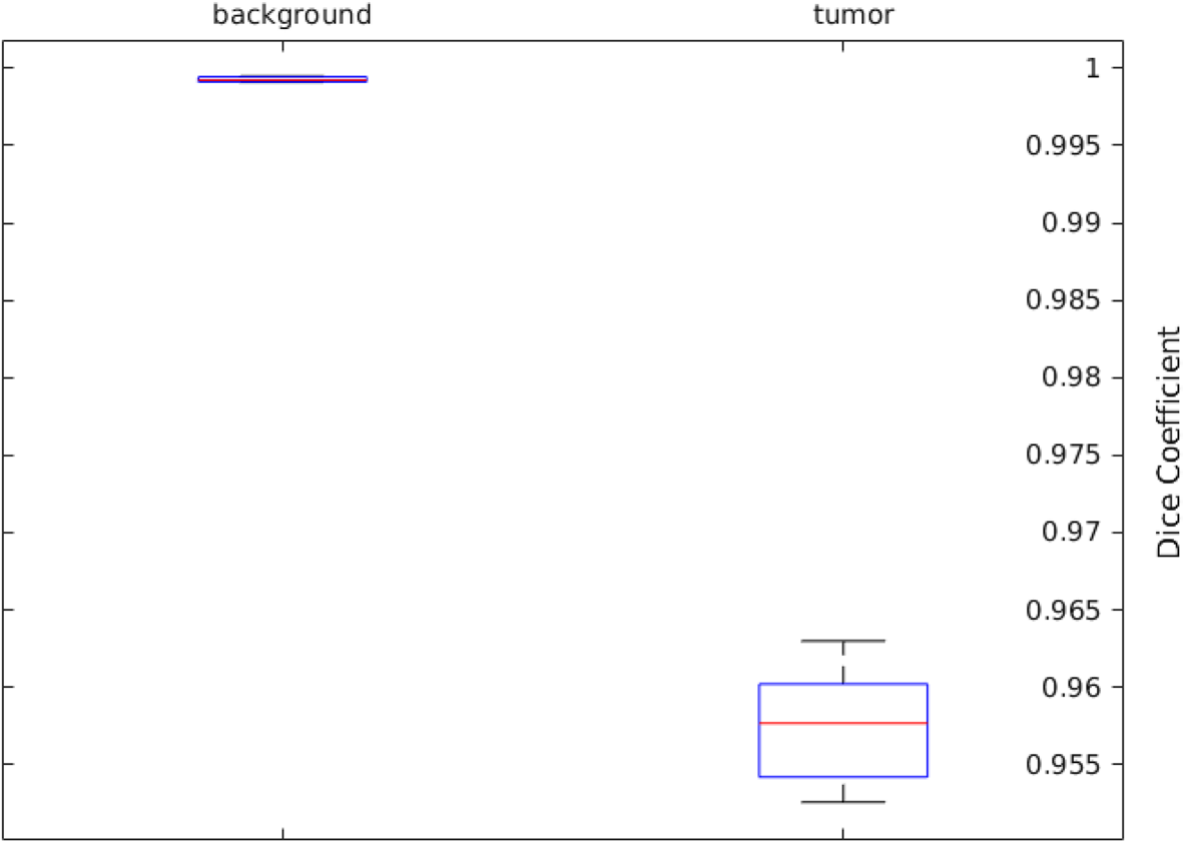
Dice Accuracy Plot of 3D U-NET Trained Network

The plot above in Fig. 11 foresees statistics about the Dice scores across the set of test volumes, while Fig. 10 gives us comparative view labeled ground truth with network prediction. The red lines in the plot above shows the median Dice value for the classes. The upper and lower bounds of the blue box indicate the 25th and 75th percentiles, respectively. Black whiskers extend to the most extreme data points not considered outliers.”

## Conclusions

We developed a fully automatic cloud-based 3D-UNet architecture for semantic segmentation on brain tumor (BRATS) dataset. Our method proves to be the most accurate cloud-based deep learning brain tumor segmentation method with a distinctive dice score of 95%. This study practices divergence of the 3-D U-Net network in which the preliminary sequences of convolutional layers (CL) are intermixed with max pooling layers. Using cloud computing has several benefits. It reduces computational cost as this network is accessible all over the globe. It only requires a stable internet connection and a terminal device for accessibility. We ran the experiment using Microsoft Azure Cloud Virtual Machine as it provides a low latency, high-throughput network interface optimized for tightly coupled parallel computing workloads. A CUDA Capable GPU is required for performing semantic segmentation of the image volumes. For intensive graphic workload like high-end remote visualization, deep learning, and predictive analytics, N-series virtual machines are ideal. NC-series virtual machines feature the NVIDIA Tesla accelerated platform K80 GPU which dramatically lowers data center costs by delivering exceptional performance with fewer, more powerful cloud servers.

## Data Availability

Dataset is freely available and can be downloaded from http://medicaldecathlon.com/. All data were made available online under Creative Commons license CC-BY-SA 4.0, allowing the data to be shared or redistributed in any format and improved upon, with no commercial restrictions. Under this license, the appropriate credit must be given, with a link to the license and any changes noted. The images can be redistributed under the same license.
